# The Role and Mechanism of *Perilla frutescens* in Cancer Treatment

**DOI:** 10.3390/molecules28155883

**Published:** 2023-08-04

**Authors:** Shicong Huang, Yi Nan, Guoqing Chen, Na Ning, Yuhua Du, Doudou Lu, Yating Yang, Fandi Meng, Ling Yuan

**Affiliations:** 1 College of Pharmacy, Ningxia Medical University, Yinchuan 750004, China; 20220710294@nxmu.edu.cn (S.H.); 20080011@nxmu.edu.cn (Y.N.); 20220720321@nxmu.edu.cn (G.C.); 20220720319@nxmu.edu.cn (N.N.); 20210710269@nxmu.edu.cn (Y.D.); 2 Key Laboratory of Ningxia Ethnomedicine Modernization, Ministry of Education, Ningxia Medical University, Yinchuan 750004, China; 3 Clinical Medical School, Ningxia Medical University, Yinchuan 750004, China; 20220130063@nxmu.edu.cn; 4 Institute of Traditional Chinese Medicine, Ningxia Medical University, Yinchuan 750004, China; 20210410204@nxmu.edu.cn (Y.Y.); 20220410212@nxmu.edu.cn (F.M.)

**Keywords:** *Perilla frutescens*, active ingredient, tumor, mechanism

## Abstract

*Perilla frutescens* is an annual herb of the Labiatae family and is widely grown in several countries in Asia. *Perilla frutescens* is a plant that is used medicinally in its entirety, as seen in its subdivision into perilla seeds, perilla stalks, and perilla leaves, which vary more markedly in their chemical composition. Several studies have shown that *Perilla frutescens* has a variety of pharmacological effects, including anti-inflammatory, antibacterial, detoxifying, antioxidant, and hepatoprotective. In the absence of a review of *Perilla frutescens* for the treatment of cancer. This review provides an overview of the chemical composition and molecular mechanisms of *Perilla frutescens* for cancer treatment. It was found that the main active components of *Perilla frutescens* producing cancer therapeutic effects were perilla aldehyde (PAH), rosmarinic acid (Ros A), lignan, and isoestrogen (IK). In addition to these, extracts of the leaves and fruits of *Perilla frutescens* are also included. Among these, perilla seed oil (PSO) has a preventive effect against colorectal cancer due to the presence of omega-3 polyunsaturated fatty acids. This review also provides new ideas and thoughts for scientific innovation and clinical applications related to *Perilla frutescens*.

## 1. Introduction

Cancer is one of the world’s leading diseases in terms of mortality [1], the second leading cause of death, and a major public health problem worldwide [2,3]. As of 2019, prostate cancer, colorectal cancer, liver cancer, and lung cancer are the four types with the highest incidence and mortality rates in men, while breast cancer and cervical cancer are the most common among female cancer patients, according to related research studies [4]. The incidence and mortality of cancer in different regions are significantly different [1,3], which is closely related to the factors leading to cancer occurrence. There have been many literature reports on the risk factors for cancer, including diet, lifestyle, family genetic history, ionizing radiation, and other nine important categories [2,5]. In addition to the more common cancer treatment methods such as surgery, chemotherapy, and radiotherapy [6], the current effective cancer treatment methods also include gene therapy [7,8] and immunotherapy [9].

Based on the existence of drug resistance and toxicity, scientists are actively looking for herbs or plant metabolites that can have anticancer potential [10,11]. Numerous studies have shown that chemicals derived from plants can have preventive and therapeutic effects on cancer [12]. Flavonoids [13,14], natural phenolic compounds from herbs and dietary plants [15], polyphenols and their compositions [16,17], and dietary unsaturated fatty acids [18] all have preventive and anti-tumor effects. Bioactive compounds derived from plants, such as tanshinone, astragaloside, berberine, ginsenoside, and matrine, can inhibit tumor growth, metastasis, and invasion by regulating the expression of abnormal miRNA and further affect tumor progression, the microenvironment, and drug resistance related to various cancers [19,20,21].

**Figure 1:**
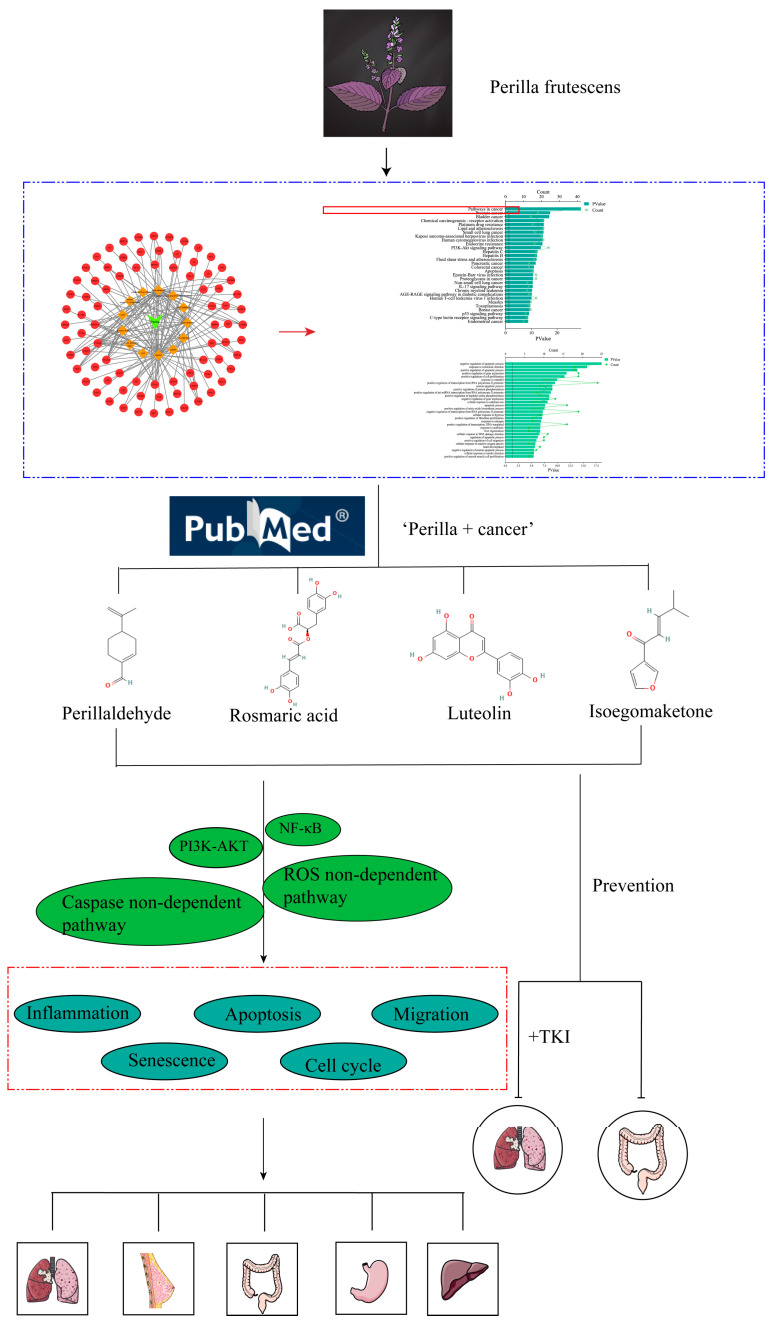
Flowchart. The antitumor effect of *Perilla frutescens* was verified by web pharmacology analysis of *Perilla frutescens*. Literature search, review, and synthesis of the literature to summarize the mechanism of action and related signaling pathways of *Perilla frutescens* as well as active ingredients against tumors. In the red box are cancer-related pathways in the KEGG pathway.

*Perilla frutescens* is an annual herb of the Labiaceae family [22]. As a common traditional herb of the same origin as medicine and food, it is widely cultivated in China, Korea, Japan, Vietnam, and other countries [23]. As a medicinal herb, *Perilla frutescens* can be subdivided into perilla seed, perilla leaf, and perilla stem. The active components of these three parts were found to have some differences after study [24,25]. Active ingredients include alkaloids [26], phenylpropane [27], terpenoids [28], polyphenols [29], flavonoids [30], anthocyanins, coumarins, carotenoids, neolignans [31,32], fatty acids, tocopherols, phytosterols, glucosides, peptides, and other related ingredients [33]. Because of the diversity of its active ingredients, *Perilla frutescens* has a wide range of pharmacological effects. According to relevant studies on *Perilla frutescens*, its pharmacological effects mainly include insecticidal effects [34], anti-allergic effects [35,36], anti-depressant effects [37], liver protection effects [38], hair growth promotion effects [39], blood lipid lowering effects, neuroprotective effects [40], anti-inflammatory effects [26], antioxidant effects [41], anticancer effects [42], and anti-tumor and antibacterial effects [43]. This review mainly summarizes the anti-cancer effect of *Perilla frutescens* systematically and provides ideas for the treatment of various tumors. The specific process is shown in Figure 1.

## 2. Network Diagram of Anti-Tumor Effect of *Perilla frutescens*

**Figure 2:**
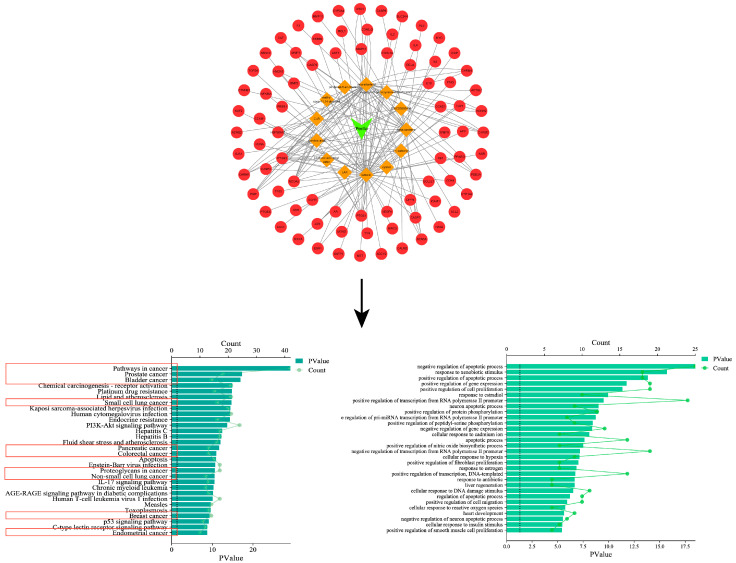
*Perilla*-active composition target plot and GO and KEGG analyses. In the red box are the signaling pathways associated with various types of cancer in the KEGG enrichment analysis.

In order to confirm the anti-tumor effect of *Perilla frutescens*, a network pharmacological analysis of *Perilla frutescens* was carried out based on its multi-component, multi-target, and multi-level properties. According to OD ≥ 0.3 and DL ≥ 0.18, 13 active components (see Supplementary File S1 for details) and 144 related proteins were screened from the TCMSP database (https://old.tcmsp-e.com/tcmsp.php (accessed on 11 May 2023)). The obtained protein name was entered in the Multiple Proteins section of the STRING database (https://cn.string-db.org (accessed on 12 May 2023)), and the Homo sapiens species was selected. Download the obtained gene name and match it with the protein name. Topological network maps of the 13 active components and 144 corresponding genes were constructed using Cytoscape software 3.9.1 (see Supplementary File S2 for details). In the figure, the plant name is the green module, the orange template is the active ingredient, and the red module is the corresponding gene. KEGG and GO analysis was performed on 144 target genes through the DAVID database (https://david.ncifcrf.gov (accessed on 12 May 2023)). The KEGG results showed that nine of the top 30 signaling pathways were associated with cancer. GO analysis showed that it was closely related to apoptosis, migration, proliferation, and other related processes (see Supplementary File S3 for details). It mainly includes a series of cancer-related signaling pathways, such as lung endometrial cancer, breast cancer, non-small cell lung cancer, pancreatic cancer, gallbladder cancer, and bladder cancer, as shown in Figure 2. These signaling pathways demonstrate that the relevant active components of *Perilla frutescens* can produce anti-tumor effects through these cancer-related pathways.

## 3. Active Ingredients

**Figure 3:**
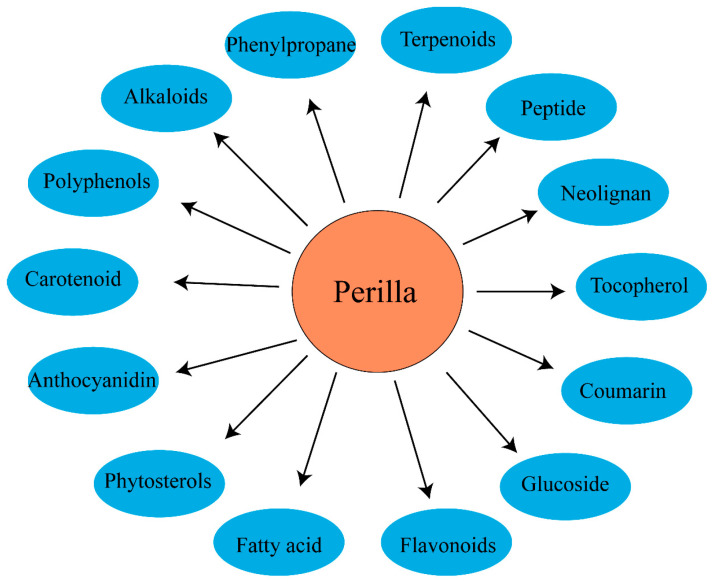
The chemical classes of *Perilla frutescens* compounds.

As an important annual herb in the *Labiaceae* family, *Perilla frutescens* has rich chemical constituents and biological functions and is widely used in food and medicine fields [44]. Related studies have found that the active components of *Perilla frutescens* can be divided into 14 main active components (see Figure 3). The detailed composition is shown in Table 1.

### 3.1. Alkaloids

Alkaloids exist in a variety of traditional Chinese herbs; for example, hyoscypane alkaloids [45] are mainly found in Solanaceae, dibenzyl isoquinoline alkaloids [46] are mainly found in the seeds and fruits of lotus, and Evodia alkaloids are mainly found in Evodia officinalis of the rutaceae [47]. In recent years, the presence of the alkaloid compound neoechinulin A in *Perilla frutescens* has been found, which can produce anti-inflammatory effects on RAW267.4 cells stimulated by lipopolysaccharide.

### 3.2. Phenylpropane

Phenylpropane compounds are secondary metabolites of plants derived from phenylalanine, an aromatic amino acid in most plants, or tyrosine in some monocotyledon plants. Some studies have found that *Perilla frutescens* collected from Taiwan contained elemin, a phenylpropanoid component, while this component was not detected in Japanese *Perilla frutescens* [48]. Some studies have found that phenylpropanoid compounds can be separated from the ethanol extract of Perilla leaves, including allyl tetramethoxybenzene, elietin, and myristin, among which elietin and myristin have been found to inhibit the production of pro-inflammatory cytokines in pneumonia in a concentration-dependent manner within a certain concentration range [49].

### 3.3. Terpenoids

Terpenoids are the most common compounds in *Perilla frutescens*, of which the monoterpenoid PAH is the main component of Perilla leaf essential oil, which can improve the in vivo function of intestinal inflammation through JNK-mediated cytokine ajay [50]. At the same time, cytoplasmic DNA-induced innate immune responses can be inhibited by inhibiting cGAS activity [51]. The triterpenoid camelliol C [52] was identified from Perilla seed species, and a series of pentacyclic triterpenes were discovered, including ursolic acid [53], oleanolic acid, corosolic acid, 3surface acid, marlinic acid, and 3-surface equine linolenic acid [54], all of which have anti-tumor effects [55].

### 3.4. Polyphenol Compounds

Common polyphenols in *Perilla frutescens* are Ros A and caffeic acid, which have been proven to have various pharmacological activities, such as anti-inflammatory [49], anti-anxiety, anti-depressive [37], hepatoprotective [56], and anticancer [42].

### 3.5. Flavonoids

Perilla leaves are composed of many types of active ingredients, but mainly flavonoids. Studies have confirmed that it acts as an anti-inflammatory agent in vivo and in vitro in specific dermatitis models [57]. The most common compound of *Perilla frutescens* flavonoids is luteolin, which has been confirmed to have anti-inflammatory, anti-itch [58], anti-allergic [59], anti-cytotoxic [60], and antibacterial [43] activities.

### 3.6. Anthocyanins, Coumarins, Carotenoids, and Neolignans

Anthocyanin pigments are the main cause of red Perilla leaves [61]. Two new lignans identified in *Perilla frutescens*, magnosalin and andamanicin, can act as inhibitors of tumor necrosis factor and nitric oxide synthesis in RAW264.7 cells induced by lipopolysaccharide [32]. Aesculin, as a coumarin, was first discovered in *Perilla frutescens* and has been found to have a certain relationship with anti-inflammatory effects [30].

### 3.7. Fatty Acids, Tocopherols, and Phytosterols

PSO is rich in active ingredients and contains a large amount of unsaturated fatty acids, which are drug and food homologies [62]. The contents of five fatty acids in Perilla seeds were identified, including palmitic acid (PA) (10.9–13.1%), stearic acid (SA) (70.3–99.11%), oleic acid (OA) (1.21–9.10%), oleic acid (1.21–9.10%), linoleic acid (LA) (2.23–4.54%), and linolenic acid (LNA) (3.75–4.100%) [63]. Omega-3 polyunsaturated fatty acids are a general term that includes alpha-linolenic acid, which is abundant in perilla oil (PO) [64]. This omega-3 polyunsaturated fatty acid has been proven to be associated with anti-inflammatory [65] and lipid metabolism disorders [66]. The intake of such dietary fatty acids can improve the intestinal flora [67] as well as intestinal inflammation and metabolic disorders in diabetic patients [68]. Tocopherols and phytosterols are rich sources of health-promoting compounds in *Perilla frutescens* [69,70]. Unsaponifiable substances, including tocopherols and phytosterols, have antioxidant and health-promoting properties [71].

### 3.8. Glucoside and Peptide

Twelve secondary metabolites isolated from perillafrutoside A, perillafrutoside B, and ten other known compounds were found in perillafrutoside A, among which perillafrutoside A can inhibit the growth of Enterococcus faecalis [72]. Monoterpene glucosides, perillosides A and C, obtained from perilli leaves, have also been found to be aldose reductases. One of the richest sources of peptides from Perilla seeds, peptides obtained from Perilla seeds can improve muscle synthesis and motor performance in mice [73]. Two novel antioxidant peptides were purified and identified from Perilla seeds to inhibit lipid peroxidation in the liver [74].

**Table 1:** Representative compounds of the main components of each class.

Active Ingredients	Species	References
Alkaloids	Neoechinulin A	[26]
Benzene propane	Eleuthero	[49]
	Myricetin	[49]
	Eugenol	[75]
Terpenoids	Perillone	[76]
	Perillaldehyde	[77]
Polyphenols	Rosmarinic acid	[78]
Flavonoids	Luteolin	[78]
	Apigenin	[29]
	Isoestradiol	[27]
	Baicalin	[79]
Anthocyanins	Malonylstilbene	[79]
	Perillin	[80]
Carotenoids	Lolliolactone	[81]
	Isoxolactone	[81]
Neolignan	Mullein	[82]
	Gooseberry	[82]
Coumarins	Heptazine	[30]
	6,7-Dihydroxycoumarin	[83]
Fatty acids	Lauric acid	[84]
	Palm oleic acid	[85]
Tocopherol	Delta-tocopherol	[86]
	Gamma tocopherol	[86]
	Beta tocopherol	[86]
	Alpha tocopherol	[86]
Glucoside	Perilla lactone A	[81]
	Perillolactone B	[81]
	Loganin	[87]
Phytosterols	Vegetable oil sterols	[86]
	Soysterol	[86]
	beta-Sitosterol	[86]

## 4. Anti-Cancer Compound Structural Formula

**Figure 4:**
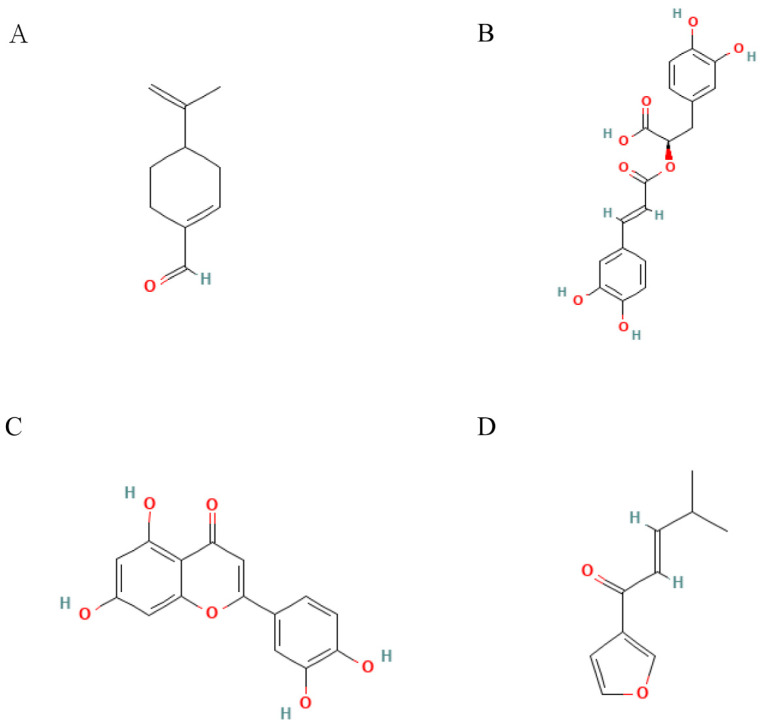
Structural formulae of representative compounds: (**A**) Perillaldehyde, (**B**) Rosmaric acid, (**C**) Luteolin, and (**D**) Isoegomaketone.

Related studies have found that *Perilla frutescens* mainly generates anti-tumor activity against liver cancer, lung cancer, and breast cancer through a series of related mechanisms such as PAH [88], IK [89], luteolin [90], Ros A [91], ethanol extract of Perilla leaves [92], Perilla extract [93], PO [94], etc. The structural formula of related compounds is shown in Figure 4.

## 5. Anti-Cancer Effect

Inducing cell apoptosis, blocking the cell cycle, reducing cell inflammation and oxidative stress, inhibiting cell metastasis, growth, and proliferation, and inducing cell senescence are the main pathways and phenotypes of the anti-tumor effects of *Perilla frutescens*, as shown in Table 2.

### 5.1. Cell Transfer

Metastases are a hallmark of cancer and cause the largest number of cancer-related deaths [95]. Epithelial-mesenchymal transformation (EMT) is a process by which epithelial cells acquire mesenchymal characteristics. In cancer, EMT is associated with tumor occurrence, invasion, metastasis, and treatment resistance [96]. Relevant studies have shown that isoproterenol (ISO) increases the migration and invasion of MDA-MB-231 human breast cancer cells and Hep3B human hepatocyte cancer cells [97]. The ethanol extract of Perilla leaf can reverse cancer cell metastasis induced by adrenergic agonists through the SRCT231F-mediated EMT pathway [92]. PAH is one of the active components of *Perilla frutescens*, which can affect prostate cancer-induced bone metastasis by inhibiting the NF-κB pathway [98].

### 5.2. Apoptosis

Apoptosis is an orderly and coordinated cellular process that occurs under physiological and pathological conditions. The mechanism of apoptosis is complex and involves many pathways. Defects can occur at any point in these pathways, leading to malignant transformation of affected cells, tumor metastasis, and anti-cancer drug resistance [99]. Ethanol extract of Perilla leaf can inhibit the growth of HCT116 and H1299 cells in a dose-dependent manner, inhibit cell colony formation, increase the G1 cell population, change nuclear morphology, and induce cell apoptosis [100]. The YAP/WW domain contains transcription factors (TAZ) that are critical for cell proliferation, survival, and self-renewal. It has also been shown to have an important carcinogenic effect on various tumors [101]. Perilla leaf extract (PLE) can induce phosphorylation of YAP/TAZ, resulting in its inactivation, and thus produce anti-tumor effects. The results suggest that PLE inhibits cell growth and increases apoptosis in breast cancer (BC) cells by inactivating YAP activity in a LATS1/2-dependent manner [102]. In the treatment of melanoma cells, IK can produce ROS, up-regulate the expression of Bax and Bcl-2, inhibit the growth of melanoma cells, and induce apoptosis.

### 5.3. Cell Cycle

Abnormal cell cycle progression is one of the basic mechanisms of tumorigenesis [103]. The *Perilla frutescens* derivative 8-hydroxy-5,7-dimethoxyflavanone (PDMF) can induce the phosphorylation of p15 and increase the expression of p21, caspase-3, and caspase-9. PDMF can trigger G2/M cell cycle arrest and apoptosis driven by p53 [104].

### 5.4. Cell Senescence

In most species, aging may induce a number of degenerative diseases characterized by a debilitating loss of tissue or cell function [105]. However, for the aging of cancer cells, its basic feature is stable proliferation arrest induced by various stressors [106]. PDMF can induce senescence in A549 human adenocarcinoma cells through the p21-p549 pathway but has no effect on normal bronchial epithelial cells [107].

### 5.5. Oxidative Stress Response and Cellular Inflammation

Tumor necrosis factor TNF-α is a major inflammatory cytokine that is particularly important in the development of tumors [108]. Endothelial microparticles are important factors in inflammation-related diseases. Studies have found that phenolic compounds contained in ethyl acetate and ethanol extracts extracted from Perilla fruit can reduce endothelial microparticles induced by TNF-α, thereby protecting endothelial cells from vascular inflammation [109]. Perilla extract can improve colitis induced by sodium dextran sulfate (DSS) in mice by inhibiting the expression of inflammation-related proteins such as COX-1. NF-κB and STAT3 are major transcriptional regulators of inflammatory signaling. Perilla extract inhibits DDS-induced NF-κB and STAT3, thereby reducing pro-inflammatory signaling [93]. Oxidative stress is a state caused by disruption of the balance between ROS production and antioxidant defense [110]. The Ros A component in PSO can reduce the production of ROS in the A549 cell line and the mRNA levels of related IL-6, IL-8, COX-2, etc., resulting in decreased expression of TNF-α induced NF-κB, JNK, MnSOD, and FOXO1 signaling pathways [91]. The ethyl acetate and ethanol extracts of *Perilla frutescens* can inhibit the production of ROS and have a protective effect on lipid peroxidation, indicating their potential to protect against oxidative stress in liver diseases [111].

### 5.6. Cell Growth

Cell growth is one of the key markers in cancer. Amp-activated protein kinase (AMPK) is associated with autophagy in unused tissues. PAH can activate AMPK by increasing phosphorylation at THr172, resulting in the increase of AMPK-related proteins such as caspase-3 and p53, resulting in increased autophagy levels and inhibiting the growth of gastric cancer [112].

### 5.7. Cell Proliferation

The abnormal proliferation of cancer cells is an important sign of a tumor and also an important reason for the expansion of cancer cells’ colonies. The PI3K/AKT signaling pathway is one of the frequently activated signaling pathways in the process of cancer, which is closely related to the occurrence and development of tumors. Studies have shown that IK isolated from Perilla extract can inhibit liver cancer (HCC) tumor proliferation by inhibiting pAKT levels without affecting total AKT levels and blocking the PI3K/AKT pathway [113].

**Table 2:** Antitumor effects of *Perilla frutescens* and its derivatives.

Type of Drug	Type of Cancer	Model	IC50 or Dose	Mechanism of Action	Reference
Perilla frutescens leaf extract	Colon cancer	HCT116 human colon cancer cells	Dose: 87.5–350 μg/mL	Inhibits the growth, colony formation, and adhesion of human colon and lung cancer cells and the migration of human lung cancer cells.	[100]
	Lung cancer	H1299 human non-small cell lung cancer cells	Dose: 87.5–350 μg/mL		
Perilla frutescens leaf extract	Triple negative breast cancer	HEK293A, MDA-MB-231, MCF10A and BT549 cells	HEK293A IC50: 584.3 μg/mL MDA-MB-231 IC50: 268.9 μg/mL MCF10A IC50: 650.8 μg/mL BT549 IC50: 307.1 μg/mL	Increased YAP phosphorylation and reduced YAP-TEAD-mediated transcriptional activity.	[102]
IK	Prostate cancer	RC-58T/h/SA#4 cells	Dose: 10–200 ng/mL	Enhancement of tumor necrosis factor-related apoptosis-inducing ligands (TRAIL)-mediated apoptosis through upregulation of DR5 by an ROS-independent pathway.	[89]
Perillaldehyde	Prostate cancer	RAW264.7 and PC-3 cells	Dose: 0.5–5 μM	Activation of the NF-κB pathway of nuclear factor-κB ligands and receptor activators to inhibit cancer cell-induced osteoclast formation.	[98]
IK	Liver cancer	Huh-7 and Hep3B cells and nude mouse models of hepatocellular carcinoma	Dose: 10 nmol/L	Significantly inhibited cell viability and xenograft tumor formation in HCC cells and inhibited AKT phosphorylation, but not AKT and p38 expression.	[113]
Perillaldehyde	Stomach cancer	MFC murine-derived cells and GC9811-P human gastric cancer cells	Dose: 0.1–5 mM	PAH activates AMPK by increasing Thr172 phosphorylation and activity; PAH increases the expression of beclin-1, LC3-II, caspase-3, and p53.	[112]
PSO and Ros A	Lung cancer	A549 lung adenocarcinoma cells	Dose PSO: 0–400 μg/mL Dsoe Ros A: 0–40 μg/mL	PSO and Ros A scavenge TNF-α induced ROS levels, resulting in reduced expression of MnSOD, FOXO1, NF-κB, and JNK signaling pathways.	[91]
PO	Breast cancer, colon cancer	Female SD rats	Dose: 10%PO	Alpha-linolenic acid-rich PO diet inhibits the development of breast, colon, and kidney tumors.	[114]
PDMF	Lung cancer	Human lung adenocarcinoma A549 cells	Dose: 30–75 μg/mL	Triggering p53-driven G2/M cell cycle arrest and apoptosis.	[104]
PDMF	Lung cancer	A549 human lung adenocarcinoma cells		Activation of the p21-p549 pathway in A53 cells; p53 is particularly important for cellular senescence	[107]
Ethanolic extract of Perilla frutescens (EPF)	Liver cancer	Human hepatocellular carcinoma HuH7 cells	IC50: 3.43 mg/mL	Protective effect of ethanol extract on the production of reactive oxygen species and lipid peroxidation in FeCl3–induction of HuH7 cells in a dose-dependent manner	[111]
Perilla frutescens leaf extract	Skin tumors	-	Dose: 0.05%	Significant reduction in tumor incidence and diversity.	[115]
IK	Melanoma	B16 melanoma cells	Dose: 10–100 μM	IK-induced apoptosis involves the production of ROS and the upregulation of Bax and Bcl-2 expression, leading to the release of cytochrome c and AIF. IK inhibits melanoma cell growth and induces apoptosis through the activation of ROS-mediated cysteinase-dependent and non-dependent pathways.	[116]
Perilla extract	Liver cancer	Human hepatocellular carcinoma HepG2 cells	Dose: 105 μg/mL	Expression of a large number of apoptosis-related genes is regulated in a time-dependent manner.	[42]
Perilla extract	Skin cancer	Two-stage skin carcinogenesis model in mice	Dose: 2.0 mg/mice	Part of the anti-cancer effect of perilla extract is due to RA through two separate mechanisms: inhibition of the inflammatory response and scavenging of reactive oxygen radicals.	[117]
PO	Liver cancer	Diethylnitrosamine (DEN)-induced hepatocellular carcinoma in male F344 rats	Dose: 5%	PO enriched with n-6 and n-3 PUFA altered the membrane fatty acid composition of the liver and inhibited the development of hepatocellular carcinoma in rats.	[118]
Luteolin	Colon cancer	HT-29 human colon cancer cells	Dose: 0–60 μmol/L	By activating caspase-3, -7, and -9, the cleavage of poly (ADP-ribose) polymerase was enhanced, the expressions of p21 (CIP1/WAF1), survivin, Mcl-1, Bcl-x(L), and Mdm-2 were decreased, and the activities of cyclin-dependent kinase (CDK)4 and CDK2 were inhibited.	[90]
PO	Breast cancer	PhIP-induced mammary carcinogenesis model in rats	Dose: 0.1%	CFA-P may retard the development of PhIP-induced breast tumors, inhibit the formation of PhIP-DNA adducts, and reduce breast carcinogenesis in the context of post-initiation inhibition of cell proliferation.	[119]
IK	Colon cancer	DLD1 colon cancer cells	Dose: 10–100 μM	IK treatment led to the cleavage of caspases-3, -8, and -9 in a dose- and time-dependent manner. IK treatment also led to cleavage of Bid and translocation of Bax. IK induced apoptosis via cystathione-dependent and caspase-non-dependent pathways in DLD1 cells.	[120]
IK	Melanoma	SK-MEL-2 human melanoma Cells	Dose: 100 μM	IK-induced ROS production regulated cell growth inhibition and induced apoptosis through cysteinase-dependent and non-independent pathways by modulating PI2K/AKT signaling in SK-MEL-3 cells. Reduced protein levels of Bax and cytochrome c as well as PARP cleavage, while protein levels of Bcl-2 were increased.	[121]
Ros A	Liver cancer	Hep G1 human liver cancer cells	IC50: 50 μM	Ros A dose-dependently attenuated aflatoxin- and hectoroxin-induced ROS production and inhibition of DNA and protein synthesis. Similarly, prevention of apoptosis by reduction of DNA fragmentation and inhibition of cysteinase-3 activation.	[122]
EPF	Liver cancer	MDA-MB-231 human breast cancer cells	Dose: 2.5–10 μg/mL	EPF inhibits the ability of adrenergic agonists to promote cancer cell metastasis by inhibiting Src-mediated EMT.	[92]
	Breast cancer	Hep3B human hepatocellular carcinoma cells	Dose: 25–100 μg/mL		

## 6. Summary of Anticancer Mechanism

**Figure 5:**
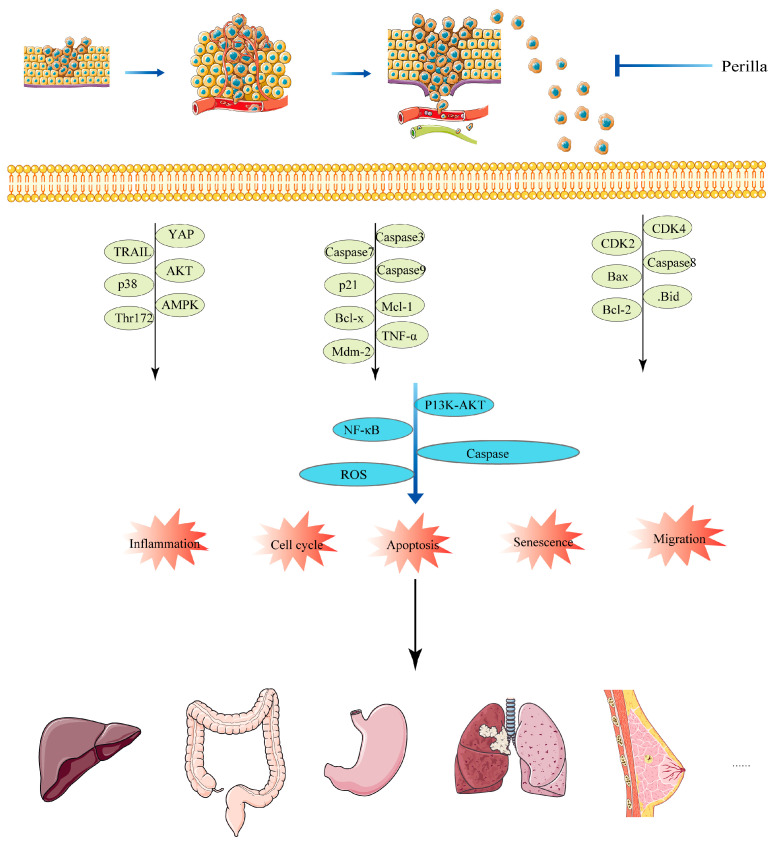
Anticancer mechanism of *Perilla frutescens*.

*Perilla frutescens* and its active components or derivatives mainly produce anti-tumor effects on cell growth, proliferation, inflammation, cycle, apoptosis, and metastasis through ROS, NF-κB, PI3K/AKT, JNK, and other pathways, as shown in Figure 5.

## 7. Preventative Effects

Relevant studies have shown that unsaturated fatty acids can be used as adjuvant therapeutic agents in cancer treatment [123]. Omega-3 polyunsaturated fatty acids (PUFAs) are considered immune nutrients and are commonly used in the nutritional treatment of cancer patients due to their rich biological effects [124]. The intake of such dietary oils is particularly important for human health. PO is a complex of unsaturated fatty acids from Perilla. PO is rich in the omega-3 polyunsaturated fatty acid alpha-linolenic acid, which can effectively reduce the risk of colon cancer [125,126] Table 3.

**Table 3:** The preventive effect of perilla and its derivatives on tumors.

Composition	Cancer	Models	IC50 or Dose	Conclusion	Mechanisms	References
PDMF	Lung cancer	A549 human adenocarcinoma of the lung	Dose: 10–125 μM	PDMF and anti-cancer tyrosine kinase inhibitors (TKI) synergistically inhibit the proliferation of A549 cells.	Synergy	[127]
PO	Colon cancer	Female F3 rats	Dose: 9%, 32%, 40%.	The relatively small amount of PO, accounting for 25% of total dietary fat, may have a significant beneficial effect in reducing the risk of colon cancer.	Preventive role	[125]
PO	Colon Cancer	Male F344 rats	Dose: 3%, 6%, 12%	PO significantly reduced ras expression and AgNORs count (a biomarker of cell proliferation) in colonic mucosa. A significant increase in n-3 polyunsaturated fatty acids in the membrane phospholipid fraction and a decrease in PGE2 levels were observed in the colonic mucosa of rats fed with PO.	Preventive role	[94]
PO	Colon cancer	Male F344 rats	Dose: 3%, 12%	β-Carotene plus PO also inhibited the number of silver-stained nucleolar organizer regions and the expression of ras mRNA (a biomarker of cell proliferation) in the colonic mucosa.	Synergy, preventive role	[128]
PO	Colon cancer	Male F20 rats	Dose: 10%, 20%	Dietary PO significantly inhibits the development of small bowel and colon tumors in APC (min) mice.	Preventive role	[129]

## 8. Summary and Outlook

Cancer is a kind of malignant disease that is difficult to treat, not only because of its diversification in proliferation and metastasis but also because cancer cells have strong adaptability [130]. Chinese herbs are an effective source of adjuvant cancer treatment and have been found to treat or prevent cancer in a variety of ways. Relevant studies have shown that various plant extracts and plant active ingredients can activate various pathways in cancer cells, including apoptosis [131]. Phenolic compounds extracted from herbs can inhibit or weaken the occurrence, progression, and metastasis of cancer [132]. Artemisinin and its derivatives have the therapeutic potential to induce iron death in cancer cells [133]. Many studies have shown that plants such as garlic, olives, and pomegranates are effective in preventing colon cancer [134].

Prior to writing this review, relevant information was consulted. Related reviews were searched in PubMed with the keywords “*Perilla frutescens,*” “*Perilla frutescens* and caner,” “*Perilla frutescens* and carcinoma,” “*Perilla frutescens* frutescens,” and “*Perilla frutescens* and tumor.” Few reviews were found in the last 5 years, and none were related to cancer treatment. Among the relevant reviews that have been reviewed, there are three related to the pharmacological and phytochemical effects of *Perilla frutescens*, which mainly summarize the phytomedicinal, ethnobotanical, phytochemical, and pharmacological effects of *Perilla frutescens* [24,135,136]. Three reviews were related to the active components of *Perilla frutescens*, namely, perillone and IK [137], Ros A [138], and PAH [88]. The review of Ros A gives an overview of its anti-cancer potential, but Ros A is derived from a variety of herbs, including rosemary and *Perilla frutescens*.

In this review, the active components of *Perilla frutescens* were summarized according to the relevant literature and the reading summary of the literature. The anticancer effects of related targets were demonstrated by biogenic analysis. The molecular mechanism and preventive effect of the components of *Perilla frutescens* with anticancer activity, including PAH, Ros A, luteolin, PO, etc. This review also gave a general description of *Perilla frutescens*’s treatment of cancer-related phenotypes, such as cell proliferation, cell metastasis, cell cycle, etc. It was found that *Perilla frutescens* mainly targeted the cellular inflammation and oxidative stress responses of cancer cells as the main targets to produce anticancer activity. In the process of writing the review, it was found that there were no relevant studies on the attenuated and synergistic effects of *Perilla frutescens* as an adjuvant therapy for cancer. *Perilla frutescens* contains many kinds of effective components, but there are few anticancer studies on PAH, PSO, perillone, and so on, which are unique to *Perilla frutescens*, and most studies focus on Ros A and IK.

Through the literature review, it was found that *Perilla frutescens* has a unique active ingredient, PO, which can prevent cancer. Through the systematic summary of PO, it was found that it is rich in omega-3 polyunsaturated fatty acids, which can have a positive effect on human health and prevent the occurrence of some diseases. Starting from this basis, according to these properties of PO, health care products and drugs can be developed to improve the quality of life, prevent major diseases, and make significant contributions to human health.

## Data Availability

All data generated or analyzed during this study are included in this paper, and further inquiries can be directed to the corresponding author (E-mail: 20080017@nxmu.edu.cn).

## Supplementary Materials

The following supporting information can be downloaded at: https://www.mdpi.com/article/10.3390/molecules28155883/s1. Supplementary File S1: The 13 active components of *Perilla frutescens* and their corresponding OB and DL values. Supplementary File S2: The protein and gene names corresponding to 13 active components of *Perilla frutescens*. Supplementary File S3: is titled KEGG and GO enrichment analysis results.
