# Supplementary material for: The Role and Mechanism of Perilla frutescens in Cancer Treatment
Source: Molecules. 2023 Aug 4;28(15):5883. doi: 10.3390/molecules28155883 (PMC10421205; doi:10.3390/molecules28155883)
Supplement: Supplementary file 1 [file molecules-28-05883-s001.zip › Supplementary File S1.pdf]

Table S1：The 13 active components of Perilla frutescens and their corresponding OB and DL values.

Mol ID	Molecule Name	OB(%)	DL
MOL000006	luteolin	36.16	0.25
MOL000358	beta-sitosterol	36.91	0.75
MOL000492	(+)-catechin	54.83	0.24
MOL000953	CLR	37.87	0.68
MOL001749	ZINC03860434	43.59	0.35
MOL001771	poriferast-5-en-3beta-ol	36.91	0.75
MOL002773	beta-carotene	37.18	0.58
MOL005030	gondoic acid	30.7	0.2
MOL006202	LAX	44.11	0.2
MOL006209	cyanin	47.42	0.76
MOL006210	eugenyl-β-D-glucopyranoside(cirtrusinc)	40.52	0.23
MOL007179	Linolenic acid ethyl ester	46.1	0.2
MOL007514	methyl icosa-11,14-dienoate	39.67	0.23
